# Stereotactic Body Radiotherapy as an Effective Treatment for Pancreatic Cancer

**DOI:** 10.7759/cureus.38255

**Published:** 2023-04-28

**Authors:** Pervin Hurmuz, Mustafa Cengiz, Gokhan Ozyigit, Sezin Yuce Sari, Alper Kahvecioglu, Caglayan Selenge Beduk Esen, Suayib Yalcin, Faruk Zorlu

**Affiliations:** 1 Radiation Oncology, Hacettepe University Medical School, Ankara, TUR; 2 Medical Oncology, Hacettepe University Medical School, Ankara, TUR

**Keywords:** pancreas tumors, pancreatic head adenocarcinoma, fractionated stereotactic radiotherapy, stereotactic ablative radiation, unresectable pancreatic cancer

## Abstract

Background

Stereotactic body radiotherapy (SBRT) allows the delivery of an ablative radiation dose to the tumor with minimal toxicity. Although magnetic resonance imaging (MRI)-guided SBRT appears to be a promising approach in the modern era, X-ray image-guided SBRT is still used worldwide for pancreatic cancer. This study aims to evaluate the results of X-ray image-guided SBRT in patients with locally advanced pancreatic cancer (LAPC).

Methodology

Medical records of 24 patients with unresectable LAPC who underwent X-ray image-guided SBRT between 2009 and 2022 were retrospectively evaluated. SPSS version 23.0 (IBM Corp., Armonk, NY, USA) was utilized for all analyses.

Results

The median age was 64 years (range = 42-81 years), and the median tumor size was 3.5 cm (range = 2.7-4 cm). The median total dose of SBRT was 35 Gy (range = 33-50 Gy) in five fractions. After SBRT, 30% of patients showed complete and 41% showed partial response, whereas 20% had stable disease and 9% had progression. Median follow-up was 15 months (range = 6-58 months). During follow-up, four (16%) patients had local recurrence, one (4%) had a regional recurrence, and 17 (70%) had distant metastasis (DM). The two-year local control (LC), local recurrence-free survival (LRFS), overall survival (OS), and DM-free survival (DMFS) rate was 87%, 36%, 37%, and 29%, respectively. In univariate analysis, a larger tumor size (>3.5 cm) and higher cancer antigen 19-9 level (>106.5 kU/L) significantly decreased the OS, LRFS, and DMFS rates. No severe acute toxicity was observed. However, two patients had severe late toxicity as intestinal bleeding.

Conclusions

X-ray image-guided SBRT provides a good LC rate with minimal toxicity for unresectable LAPC. However, despite modern systemic treatments, the rate of DM remains high which plays a major role in survival.

## Introduction

The prognosis of pancreatic cancer is quite poor among all gastrointestinal system cancers. It generally affects the elderly and fragile population, and more than 66% of newly diagnosed patients are over 65 years of age [1]. Radical surgery is the only curative treatment approach for pancreatic cancer; however, most patients are not eligible for surgery. In addition, other treatments such as chemotherapy (CHT) and radiotherapy (RT) are needed in high-risk patients even after surgery or as a definitive treatment option for inoperable and unresectable patients.

Definitive treatment of unresectable locally advanced pancreatic cancer (LAPC) includes neoadjuvant systemic therapy followed by chemoradiotherapy (CRT) for patients who still have an unresectable disease [2]. Definitive conventionally fractionated CRT is applied with fraction doses of 1.8-2 Gy in 25-28 fractions which continues for approximately six weeks. Stereotactic body radiation therapy (SBRT), on the other hand, is a promising RT technique in patients with LAPC which can apply high ablative doses to the tumor in fewer fractions, allowing the surrounding healthy tissues to receive lower doses. Due to these advantages, SBRT has become an increasingly popular treatment option for patients with LAPC and has been studied in many clinical studies. In a meta-analysis published by Tchelebi et al. [3] in 2019, it was stated that SBRT might contribute to overall survival (OS) compared to conventional CRT, and the toxicity is also quite low.

With the current developments in technology, the precision of radiation dose delivery in SBRT has been increasing. However, X-ray image-guided SBRT, used worldwide, is still a preferred approach when magnetic resonance imaging (MRI) guidance is not eligible. In this study, we retrospectively analyzed the oncological outcomes and toxicity profile of patients who underwent X-ray image-guided SBRT for LAPC in a single comprehensive cancer center.

## Materials and methods

Patient population

Medical records of patients who underwent SBRT for unresectable LAPC in our institution between March 2009 and March 2022 were evaluated in this retrospective analysis. Patients who had metastatic lymph nodes, treated with conventionally fractionated CRT or a previous abdominal RT, patients without treatment-related toxicity information or radiological response evaluation imaging after SBRT, and medically inoperable patients with resectable tumors were excluded from the study. After these exclusions, a total of 24 patients were considered for inclusion. This study was conducted in compliance with the principles of the Helsinki Declaration.

Stereotactic body radiation therapy

**Figure 1:**
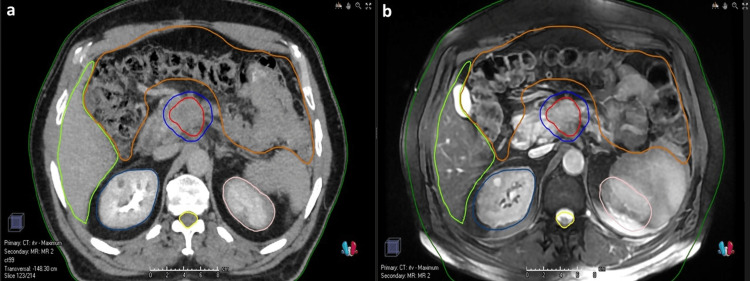
Target volumes of a patient treated with CT-based SBRT. Fusion of simCT (a) and MRI (b) are seen. Navy blue: PTV (33 Gy/5 fractions); red: ITV (40 Gy/5 fractions, SIB); dark green: body; light green: liver; brown: bowel; pink: left kidney; blue: right kidney; yellow: spinal cord. CT = computed tomography; SBRT = stereotactic body radiation therapy; simCT = simulation computed tomography; MRI = magnetic resonance imaging; PTV = planning target volume; ITV = internal target volume; SIB = simultaneous integrated boost

The respiratory motion management techniques were either tumor tracking or internal target volume (ITV)-based approach. For patients without fiducial markers, a four-dimensional computed tomography (4DCT) was performed for RT simulation using a 2.5 mm slice thickness with an intravenous contrast agent and appropriate immobilization devices in the supine position. MRI and positron emission tomography-computed tomography (PET/CT) images were fused with 4DCT images for the gross tumor volume (GTV) and subsequent ITV delineation. Separate GTVs were delineated per patient according to simulation CT images achieved in different respiration phases. Eventually, ITV was delineated by the fusion of all these images. In patients with fiducial markers, 4DCT was not performed and only GTV was delineated. The planning target volume (PTV) was delineated by adding 3-5 mm margins to the GTV for patients with fiducial markers and to the ITV for patients without fiducial markers. Elective nodal irradiation was not performed for any patient. Our institutional prescribed doses for PTV are consistent with the American Society for Radiation Oncology (ASTRO) recommendations which are between 33 Gy and 40 Gy in five fractions [4]. The dose constraints applied in our clinic for the organs at risk (OAR) are presented in Table 1. Dose prescriptions were defined based on the location and size of the tumor and its proximity to the OARs. For eligible patients, the prescribed dose to the GTV was escalated with a simultaneous integrated boost (SIB) technique. The biologically equivalent dose (BED) values were calculated as Total radiation dose × (1 + [Dose per fraction ÷ α/β]). Target volumes of a patient treated with X-ray image-guided SBRT of 33 Gy (BEDα/β10Gy = 54.8 Gy) to the PTV and 40 Gy (BEDα/β10Gy = 72 Gy) to the ITV in five fractions with the SIB technique is presented in Figure 1. SBRT treatments were delivered via Synergy® (Elekta Inc. Sweden), Novalis® (BrainLAB, Germany), or Cyberknife® (Accuray Inc., Sunnyvale, CA). IGRT was applied using a daily cone-beam CT (CBCT) or kilovoltage (kV) radiography-based imaging in all fractions.

**Table 1:** Dose constraints for organs at risk at five fractions of stereotactic body radiotherapy. Dx cc = dose received by x cc of organ volume; Dmean = dose received by the mean value of organ volume

Organ at risk	Dose constraint
Duodenum	D0.1 cc <33 Gy (optimal), <35 Gy (mandatory)
Kidney	Dmean <10 Gy D200 cc <17.5 Gy
Liver	D700 cc <15 Gy
Stomach	D0.1 cc <33 Gy (optimal), <35 Gy (mandatory) D10 cc <25 Gy D50 cc <12 Gy
Small bowel	D0.1 cc <30 Gy (optimal), <35 Gy (mandatory) D10 cc <25 Gy

Follow-up and toxicity

Following SBRT, all patients were monitored every three months during the first two years, every six months for the next three years, and annually thereafter. Radiological treatment response evaluation and local-regional recurrence (LRR) were defined according to the Response Evaluation Criteria in Solid Tumors (RECIST) criteria [5]. For toxicity assessment, institutional patient files and the electronic medical system of our hospital were evaluated. Common Terminology Criteria for Adverse Events (CTCAE) v4.0 was used to assess acute and late side effects.

Statistical analysis

SPSS version 23.0 (IBM Corp., Armonk, NY, USA) was used for all statistical analyses. All time-related events were defined as from the completion of SBRT to the last follow-up, death, or recurrence, whichever came first. Kaplan-Meier estimates were used for survival analysis. Age; gender; tumor size; SBRT dose; BEDα/β10Gy value; administration of neoadjuvant, concomitant, and adjuvant CHT; type of CHT regimen; cancer antigen 19-9 (CA 19-9) level; systemic inflammation index (SII); and neutrophil-lymphocyte ratio (NLR) before SBRT were defined as covariates for survival. The SII levels were found by multiplying the platelet count with the NLR [6]. We categorized the CA 19-9, SII, and NLR levels into two using their median values for statistical analyses. A p-value of <0.05 was considered statistically significant.

## Results

Patient, tumor, and treatment characteristics

The patient, tumor, and treatment characteristics are shown in Table 2. The median age of the patients was 68 years (range = 42-81 years). The majority of patients were male. The staging was performed radiologically with a combination of MRI and PET-CT in all patients. Biopsy was available in 95% of patients, and the tumor histology was ductal adenocarcinoma in all. No patients had DM at diagnosis. The median number of neoadjuvant FOLFIRINOX was six cycles (range = 4-6 cycles), and the median number of neoadjuvant gemcitabine was five cycles (range = 4-6 cycles). Neoadjuvant CHT could not be applied due to comorbidities and/or performance in eight patients.

**Table 2:** Patient, tumor, and treatment characteristics. CHT = chemotherapy; FOLFIRINOX = leucovorin calcium (folinic acid), fluorouracil, irinotecan hydrochloride, and oxaliplatin; SII = systemic inflammation index; NLR = neutrophil to lymphocyte ratio; SBRT = stereotactic body radiation therapy; BED = biologically effective dose; PTV = planning target volume

Characteristics	Number (%)
Gender	
Male	16 (66)
Female	8 (34)
Stage (TNM)	
IIA	4 (16)
IIB	3 (12)
III	17 (72)
Neoadjuvant CHT	
None	8 (33)
FOLFIRINOX	8 (33)
Gemcitabine	8 (33)
Response to neoadjuvant CHT	
Partial	7 (43)
Stable	9 (57)
Prognostic parameters	
CA 19-9 (median)	106.5 kU/L (range = 31–351 kU/L)
SII (median)	615 (range = 281–798)
NLR (median)	2.1 (range = 1.5–4)
Tumor size (median)	3.5 cm (range = 2.7–4 cm)
SBRT	
Number of fractions (median)	5 (range = 3–5)
Total PTV dose (median)	35 Gy (range = 33–50 Gy)
Concurrent capecitabine	
Yes	8 (34)
No	16 (66)
Response to SBRT	
Complete	7 (30)
Partial	10 (42)
Stable	5 (20)
Progression	2 (8)
Adjuvant CHT	
None	8 (33)
FOLFIRINOX	8 (33)
Gemcitabine	8 (33)

No patients had metastatic lymph nodes (LN) before SBRT, according to their pre-SBRT imaging, such as MRI, CT, and PET/CT, and elective nodal irradiation was not performed in any patients. The number of SBRT fractions was five in 23 patients and three in one patient. The median BEDα/β10Gy for PTV was 59.5 Gy (range = 54.8-100 Gy). No patients were operated on after SBRT. Adjuvant CHT was not applied due to medical comorbidities and performance status in eight patients.

Oncologic outcomes

The median follow-up was 15 months (range = 6-58 months). The median time of the first response evaluation after SBRT was three months (range = 3-6 months). Following SBRT, 30% of patients showed complete response, 41% showed partial response, 21% had stable disease, and 8% progressed. The one and two-year local control (LC) rate was 87% and 87%, respectively. During the follow-up, four (16%) patients had a local recurrence, one (4%) had regional recurrence (para-aortic LN), and 17 (70%) had distant metastasis (DM) (peritoneum, n = 9; liver, n = 7; lungs, n = 5; bones, n = 4).

The one and two-year overall survival (OS), local recurrence-free survival (LRFS), and DM-free survival (DMFS) rate was 82% and 37%, 73% and 36%, and 57% and 29%, respectively. Median OS, LRFS, and DMFS was 15.9 ± 3.9 months (95% confidence interval (CI) = 8.8-23.1 months), 15.1 ± 3.6 months (95% CI = 8.7-23.1 months), and 13.3 ± 2 months (95% CI = 9.4-17.2 months), respectively.

The results of the univariate analysis are in Table 3. The two-year OS, LRFS, and DMFS rates were higher in patients with pre-SBRT CA 19-9 level ≤106.5 kU/L compared to those with >106.5 kU/L and in patients with a tumor size ≤3.5 cm compared to those with >3.5 cm. The SII or NLR values before SBRT did not affect any survival rate. The administration of neoadjuvant, concomitant, or adjuvant CHT and the CHT regimen had no effect on any survival parameter. Multivariate analysis was not performed due to the small number of patients.

**Table 3:** Results of univariate analysis for two-year OS, LRFS, and DMFS. OS = overall survival; LRFS = local recurrence-free survival; DMFS = distant metastasis-free survival; NLR = neutrophil to lymphocyte ratio; SII = systemic inflammation index; SBRT = stereotactic body radiation therapy; BED = biologically equivalent dose; CHT = chemotherapy; FOLFIRINOX = leucovorin calcium (folinic acid), fluorouracil, irinotecan hydrochloride, and oxaliplatin

Variable	OS (%)	P-value	LRFS (%)	P-value	DMFS (%)	P-value
Age (years)						
≤64	50	0.164	48	0.413	40	0.280
>64	23		22		13	
Sex						
Male	44	0.525	43	0.433	30	0.945
Female	38		22		33	
Tumor size						
≤3.5 cm	58	0.042	57	0.029	39	0.019
>3.5 cm	14		10		12	
CA 19-9						
≤106.5 kU/L	58	0.006	56	0.021	50	0.019
>106.5 kU/L	12		11		0	
NLR						
≤2.1	14	0.117	12	0.100	13	0.283
>2.1	60		57		39	
SII						
≤615.5	46	0.474	45	0.291	41	0.348
>615.5	25		23		15	
SBRT dose						
≥35 Gy	46	0.536	44	0.649	38	0.621
<35 Gy	25		24		15	
BEDα/β10Gy						
≥60 Gy	43	0.325	40	0.447	37	0.871
<60 Gy	38		39		18	
Neoadjuvant CHT						
Yes	39	0.782	38	0.794	28	0.781
No	35		34		33	
Neoadjuvant CHT regimen						
Gemcitabine	38	0.899	36	0.839	25	0.945
FOLFIRINOX	40		39		31	
Concurrent CHT						
Yes	22	0.266	21	0.249	21	0.249
No	43		39		42	
Adjuvant CHT						
Yes	37	0.619	36	0.928	31	0.381
No	33		30		28	
Adjuvant CHT regimen						
Gemcitabine	41	0.177	39	0.329	34	0.483
FOLFIRINOX	39		33		27	

Toxicity

Grade 1-2 nausea and vomiting was observed in three patients during treatment. No patients experienced severe acute toxicity. However, two (8%) patients had a grade 3 late toxicity as intestinal bleeding. One of these patients received 24 Gy in three fractions, and the other received 33 Gy in five fractions of SBRT. The patient who underwent three fractions of SBRT was treated with a fiducial marker-based approach and the patient who underwent five fractions of SBRT was treated with an ITV-based approach, without a fiducial marker. The dose constraints of OARs in both patients were consistent with the recommendations [7]. Both patients underwent medical treatments such as blood transfusions and did not require any surgical intervention. We report these as treatment-related toxicity although the localization of bleeding is unknown due to the absence of endoscopic interventions at the time of bleeding. There was no toxicity-related death or local recurrence in either of them, but they succumbed to death due to metastatic disease progression.

## Discussion

In this single-center study, we evaluated the treatment outcomes in patients who received X-ray image-guided SBRT for pancreatic cancer. With a median follow-up of 15 months, we found a satisfactory LC rate with acceptable toxicity. However, the rates of DMFS and OS were still low despite the use of neoadjuvant CHT. We observed that a lower CA 19-9 level and a smaller tumor size were associated with improved survival. Our data also show that elective lymphatic irradiation may not be needed in these patients.

Although surgery is the primary treatment of pancreatic cancer, CHT ± CRT yields an important survival benefit in certain cases with high-risk factors and inoperable/unresectable disease. A conventionally fractionated RT (CFRT) schedule of five to six weeks with concomitant CHT is routinely used in the definitive or neoadjuvant setting of LAPC. However, LAPC has a high risk of DM and effective systemic treatment should begin as soon as possible. Thus, there is an increasing interest in SBRT because of its potential to minimize the delay of systemic therapy and increase the LC rate by allowing a higher BED value compared to CFRT. SBRT can be used both in the definitive and neoadjuvant setting for LAPC.

Although there is no randomized study directly comparing CFRT with SBRT, several retrospective and early prospective studies have shown that SBRT provides oncologic outcomes similar to, and in some studies, better than CFRT. A retrospective study from MD Anderson Cancer Center evaluated the efficacy of a median 36-Gy SBRT in five fractions and 50.4-Gy CFRT in 28 fractions in 104 patients [8]. The authors reported a slight OS benefit with SBRT without a statistically significant difference (median of 29.6 months vs. 24.1 months with CFRT). In another retrospective study based on the National Cancer Database (NCDB), the two-year OS rate was reported to be significantly higher with SBRT than with CFRT (21.7% vs. 16.5%; p = 0.001) [9]. Similarly, a systematic review and meta-analysis of 20 studies (nine SBRT, 11 CFRT) by Tchelebi et al. [3] showed that the two-year OS rate was significantly higher in the SBRT group than in CFRT (26.9% vs. 13.7%). The authors also reported substantially fewer acute grade 3 and higher toxicities in the SBRT arm (5.6% vs. 37.7% for CFRT) with similar rates of late toxicity.

Variable LC and toxicity rates have been reported in studies based on varying doses of SBRT. For instance, while 25 Gy in a single fraction led to disease stability, it also caused unacceptable duodenal toxicity, including intestinal bleeding and perforation [10,11]. Using three to five fractions of SBRT resulted in favorable LC rates and reduced toxicity compared to CFRT [12,13]. In our study, the one and two-year LC rates were both 87% with fractionated SBRT, and the severe late toxicity rate was 8%. Considering the features of patients with intestinal bleeding as treatment-related toxicity, a five-fraction SBRT regimen can be preferred over three fractions, and CBCT over kV radiography-based imaging for IGRT to avoid overlapping of the target volumes and OARs. Our high LC and acceptable toxicity rates support the efficacy and safety of fractionated X-ray image-guided SBRT in patients with LAPC.

SBRT has also some theoretical advantages over CFRT in addition to its high LC and low toxicity rates. One of them is SBRT allows a shorter overall treatment time, less delay for effective systemic therapy, and increased convenience. Additionally, SBRT can provide higher BED values to the tumor, and thus, has the potential to overcome the intrinsic radiation resistance of LAPC. In a study by Zhu et al. [14], BEDα/β10Gy ≥60 Gy with SBRT was associated with improved oncologic outcomes for pancreatic cancer. Our median total SBRT dose was 35 Gy in five fractions, and the median BEDα/β10Gy was 59.5 Gy. However, we did not observe an improvement in oncologic outcomes with higher BEDα/β10Gy values, probably due to the limited number of patients.

Elective nodal irradiation is not routinely recommended for SBRT due to the lack of prospective data. However, retrospective studies reported a decreased lymphatic recurrence rate with elective nodal irradiation without a significant increase in late toxicity, but it also does not affect survival [15]. Although we did not treat the lymphatics, only one patient developed a regional recurrence at the para-aortic region. As elective nodal irradiation significantly increases the sizes of target volumes, it should not be routinely treated in SBRT treatments, unless a high level of evidence is obtained in the future.

IGRT has increased the quality of treatments applied in the modern era. MRI-guided SBRT has also become an increasingly popular and exciting topic for pancreatic SBRT. MRI provides a better soft tissue visualization than CT and allows for online adaptive planning. The proximity of OARs such as the stomach and duodenum to the target volumes is an important dose-limiting factor for pancreatic SBRT, and online adaptive planning may provide dose escalation to the tumor without increasing toxicity. Several studies have shown that very high BEDα/β10Gy values can be delivered safely with in-room MR guidance. In a retrospective study involving 35 patients, the authors reported a one-year LC rate of 87% and an OS rate of 58% with 50 Gy MRI-guided SBRT in five fractions [16]. However, although a BEDα/β10Gy ≥60 Gy is recommended for pancreatic SBRT, the benefit of additional dose escalation remains unclear [14]. Considering our two-year LC rate of 87%, CT-guided SBRT may provide a similar LC rate compared to MR-guided SBRT with minimal toxicity. However, our study may have several biases due to its retrospective design and the comparison of the LC rates warrants further investigation. According to the data in hand, it is still not adequate to conclude that MR-guided SBRT should be the standard of care for pancreatic SBRT, and there is still a need for the long-term results of ongoing studies focusing on the benefit of dose escalation on oncologic outcomes. When deciding on the optimal treatment for patients, the available resources should also be considered. Our findings are important in showing that X-ray image-guided SBRT, still used worldwide, is an effective and safe treatment option for patients with unresectable pancreatic carcinoma.

Although neoadjuvant CHT has potential benefits (e.g., turning unresectable patients into resectable and increasing R0 resection rates in patients undergoing surgery), the optimal CHT regimen remains unclear. Three non-randomized phase II studies evaluating the efficacy of a neoadjuvant FOLFIRINOX regimen have shown its feasibility with high R0 resection rates [17-19]. Although the multi-agent FOLFIRINOX regimen is superior to gemcitabine monotherapy in the adjuvant setting and metastatic patients, there is no high level of evidence for its superiority in the neoadjuvant setting [20,21]. In our study, we observed that neoadjuvant CHT and the regimen had no effect on survival rates. Likewise, the use of concomitant or adjuvant CHT did not improve the oncologic outcomes, and most patients developed DM following SBRT. It is clear that more effective and less toxic systemic therapies are needed for improving systemic control in this patient population.

There are several prognostic parameters for pancreatic cancer. The CA 19-9 serum carbohydrate antigen is a tumor-associated antigen and the only Food and Drug Administration-approved prognostic marker in LAPC, and high levels of CA 19-9 predict a dismal prognosis [22,23]. In our study, the median survival of patients with a CA 19-9 level ≤106.5 kU/L was significantly longer in accordance with the literature. The SII is calculated based on the neutrophil, platelet, and lymphocyte counts, and can provide more promising prognostic information than NLR and platelet-lymphocyte radio in some cancers [24-26]. Although the precise mechanism has not been completely understood, it is considered that systematic inflammation may play a critical role in the pathogenesis and progression of cancer and predict a worse survival outcome. However, in our study, the SII value before SBRT did not affect the survival rates. CA 19-9 remains the only approved biomarker for diagnosis and response assessment for patients with pancreatic cancer with low sensitivity and specificity. Yet, SII appears to be a promising biomarker for prognosis [27-29].

This study has some limitations, such as its retrospective design, short follow-up period, suboptimal systemic therapy due to the fragility of the patient population, and the small number of patients. However, all patients diagnosed with unresectable tumors were treated in a tertiary center with a homogenous SBRT treatment protocol. Our SBRT regimens seem effective with acceptable toxicity. To introduce SBRT more in routine clinical use, there is a great need for prospective studies on dose-fractionation schedules, effects of dose escalation, appropriate timing of the multimodal treatment, toxicity profile, and safety of combination with systemic therapies.

## Conclusions

Although MRI-guided SBRT appears to be a promising approach in the modern era, X-ray image-guided pancreatic SBRT, used worldwide, is still an effective and safe treatment modality that allows a high-dose prescription to the tumor while maximizing normal tissue preservation. It provides high LC and acceptable toxicity rates for patients with unresectable pancreatic cancer. According to our study, smaller tumor sizes and lower CA 19-9 levels are associated with improved survival. However, even SBRT provides high LC, DM has a major impact on survival, and more effective systemic therapies are needed.
